# The Effectiveness of Loyalty Rewards to Promote the Use of an Internet-Based Heart Health Program

**DOI:** 10.2196/jmir.3458

**Published:** 2014-07-02

**Authors:** Sam Liu, Corinne Hodgson, Ahmad M Zbib, Ada YM Payne, Robert P Nolan

**Affiliations:** ^1^Cardiac eHealth Research UnitPeter Munk Cardiac CenterUniversity Health NetworkToronto, ONCanada; ^2^Institute of Medical ScienceFaculty of MedicineUniversity of TorontoToronto, ONCanada; ^3^Corinne S Hodgson & AssociatesBurlington, ONCanada; ^4^Digital Health and InnovationHeart and Stroke Foundation of CanadaToronto, ONCanada

**Keywords:** Internet, cardiovascular risk, prevention, rewards

## Abstract

**Background:**

Internet-based health programs have been shown to be effective in reducing risk for cardiovascular disease. However, their rates of enrollment and engagement remain low. It is currently unclear whether rewards from established loyalty programs can serve as a conditioned stimulus to improve the use of a freely available Internet-based program.

**Objective:**

The objectives of the study were to (1) examine enrollment rates and levels of engagement with the My Health eSupport program between a Conditioned Reward group and a Control group, and (2) investigate the influence of loyalty rewards and participant characteristics on levels of enrollment and program engagement.

**Methods:**

The study sample (n=142,726) consisted of individuals who were offered enrollment in an Internet-based health intervention (My Health eSupport) after completing the Heart&Stroke Risk Assessment on the Heart and Stroke Foundation website. My Health eSupport programs provided encouragement and tips for lifestyle change. This is a free, self-guided, fully automated program that proactively delivers tailored email messages at 2-week intervals based on the participant’s stage of motivational “readiness” and priority for lifestyle change. Participants in the Conditioned Reward group were offered a single exposure of 20 loyalty reward points from the Air Miles loyalty program for completing the Heart&Stroke Risk Assessment (10 reward points) and enrolling in the Internet-based program (10 reward points). Meanwhile, no rewards were given to the Control group participants. All data were collected between February 1, 2011 and February 10, 2012.

**Results:**

In total, 51.38% (73,327/142,726) of individuals in the Conditioned Reward group and 48.62% (69,399/142,726) of individuals in the Control group completed the Heart&Stroke Risk Assessment. Subsequently, significantly more individuals from the Conditioned Reward group (52.96%, 38,835/73,327) enrolled in the My Health eSupport program than Controls (4.07%, 2826/69,399). Regression analyses indicated that individuals were 27.9 times (95% CI 26.4-29.4; *P*<.001) more likely to join the My Health eSupport program when presented with loyalty rewards controlling for gender, age, education, ethnicity, employment, and number of modifiable risk factors. However, ongoing engagement level was low in both groups and it was not influenced by loyalty rewards. Instead, individuals were more likely to engage with the My Health eSupport program if they were greater than 60 years of age (OR 12.56, 95% CI 5.66-27.8; *P*<.001), were female (OR 1.27, 95% CI 1.09-1.46; *P*=.002), or had one or more modifiable risk factors (OR 1.38, 95% CI 1.31-1.45; *P*<.001).

**Conclusions:**

Our findings suggest that a single exposure of loyalty rewards may be used to encourage individuals to enroll in an Internet-based preventative health program, but additional strategies are required to maintain engagement level. Future studies need to examine the schedules of loyalty reward reinforcement on the long-term engagement level of Internet-based health programs.

## Introduction

Cardiovascular diseases (CVDs) are the number one cause of death globally. It is estimated that the number of people who die from CVDs will reach 23.3 million by 2030 [1]. Studies have shown that more than 80% of CVDs can be prevented through lifestyle changes, such as regular exercise and a healthy diet [2].

The wide adoption of Internet usage presents an incredible opportunity for delivering preventive health initiatives at a population level. In 2012, approximately 83% of Canadians had personal access to the Internet: 85% of whom live in metropolitan areas and 75% in rural areas [3]. This includes 63-78% of Canadians who are in the lowest income quartiles and 84% of Canadians between 45 and 64 years, which is an age group with increased risk for CVD. Several randomized controlled trials and meta-analyses have shown that Internet-based lifestyle interventions can help individuals improve self-care behaviors (physical activity, dietary habits) [4,5], psychological functioning (anxiety, quality of life) [6], and clinical outcomes (blood pressure, weight, blood glucose) [7-9]. However, enrollment and engagement in Internet-based health interventions remain a challenge [10-12]. This can limit the significant impact that Internet-based interventions have on health behavior change at a population level.

External rewards such as financial and loyalty rewards have been used as a strategy to increase the enrollment and engagement of behavioral interventions [13,14]. The theoretical rationale for using these external rewards to reinforce behavior change is based on both operant learning and behavioral economic theory [13,15]. The key principle of operant learning is that behavior is conditioned by contingencies that are rewarding or punishing in nature [13]. Economic theory includes the principles from operant theory and states that an individual tends to behave in ways that maximize one’s immediate reward [13,15]. According to these theories, the propensity for enrollment and sustained engagement in Internet-based programs may be enhanced by introducing external rewards. On the contrary, self-determination theory (SDT) asserts that an external reward may weaken intrinsic motivation for behavior change and this is known as the “crowding out” effect [16,17]. It is currently unclear whether a single bout of reward from a loyalty program can serve as a conditioned stimulus affecting the enrollment and engagement of a freely available Internet-based health program. An advantage of using loyalty rewards over financial ones is that it may be more sustainable as both businesses and consumers can mutually benefit. Loyalty reward programs can help businesses gain new and retain existing customers by rewarding them with loyalty “reward points”. These customers can then redeem their reward points for goods and/or services from the loyalty program. Thus, the aims of this study were to (1) examine enrollment rates and levels of engagement with the My Health eSupport program between the Conditioned Reward and the Control group, and (2) investigate the influence of loyalty rewards and participant characteristics on levels of enrollment and program engagement. A previous study reported that a single bout of financial reward (US$2.00-$20.00) could increase enrollment and response to a follow-up survey 3 months after enrollment for an Internet-based health program [18]. Therefore, we hypothesized that enrollment rates and level of engagement would be higher in the Conditioned Reward group than Controls. Results were expected to be influenced by age, ethnicity, education, gender, employment status, and the number of modifiable risk factors [11,18].

## Methods

### Overview

This was an observational study and the data was collected by the Heart and Stroke Foundation of Canada (HSFC) between February 1, 2011 and February 10, 2012. The HSFC is a non-profit public organization that aims to empower Canadians to live healthy lives free of heart disease and stroke by raising awareness of the key risk factors and encouraging and supporting them to play an active role in managing their health. Study participants were 18 years or older and provided consent for the study. All personal identifiers were removed prior to retrieving the records from the HSFC database. This study was approved by the University Health Network Ethics Board.

### Recruitment and Study Protocol

The study sample consisted of individuals who were invited to participate in an Internet-based heart health program (My Health eSupport) after completing their Heart&Stroke Risk Assessment on the HSFC website. A total of 142,726 individuals completed the Heart&Stroke Risk Assessment and were included in our analyses. This sample was comprised of two groups: Conditioned Reward vs Control. Participants in the Conditioned Reward group were recruited from the Air Miles loyalty program using a standardized recruitment email. The email described the opportunity to receive a non-cash, uniform reward of up to 20 Air Miles reward points for enrolling in the My Health eSupport HSFC program. Specifically, participants were assured of immediately receiving 10 Air Miles reward points for completing the Heart&Stroke Risk Assessment and another 10 Air Miles for enrolling in My Health eSupport. No additional rewards were given after enrollment. The Heart&Stroke Risk Assessment was a free e-tool on the HSFC website that first enabled individuals to assess their lifestyle in regard to CVD risk. It then offered enrollment to the My Health eSupport program. Enrollment in the My Health eSupport program required the participants to first complete the Heart&Stroke Risk Assessment and create a log-in ID on the HSFC website. Air Miles participants accessed the Heart&Stroke Risk Assessment and claimed the reward using a unique Web link embedded in the recruitment email. Air Miles participants who completed the Heart&Stroke Risk Assessment were assigned to the Conditioned Reward group. Participants in the Control group accessed the Heart&Stroke Risk Assessment on the HSFC website without using the unique Web link provided by the Air Miles loyalty program. Control participants did not receive any rewards for completing either program.

### Program Description

The My Health eSupport program was a free, self-guided, fully automated healthy lifestyle program that proactively delivered tailored email messages at 2-week intervals. The emails contained information on heart healthy living with links to the HSFC website. The initial email from My Health eSupport was delivered to the participants following enrollment. The email guided participants to report their stage of motivational “readiness” to adhere to Health Canada guidelines for diet (daily intake of fruit, vegetables, and restriction of dietary fat and salt), exercise (planned exercise and daily activity), and smoke-free living. Readiness for change was operationally defined for each behavior according to the conventional algorithm of Prochaska’s Transtheoretical Model [19] (Pre-contemplation, Contemplation, Preparation, Action, and Maintenance). In an effort to reinforce motivation and efficacy for self-directed change [20], subjects were then prompted to choose their priority for lifestyle change from the above-noted behaviors associated with diet, exercise, and smoke-free living. Subsequently, three email messages tailored to the individuals’ readiness and priority for lifestyle change were delivered at 2-week intervals. The assessment of readiness and priority for lifestyle change was then repeated 6 weeks later from the initial assessment.

### Measures

This paper evaluated enrollment rates and levels of engagement in the My Health eSupport program in both the Conditioned Reward and Control groups. Enrollment rate was calculated as the number of individuals who enrolled in the My Health eSupport program divided by the number of participants who completed the Heart&Stroke Risk Assessment. Two measures of engagement level with the program were calculated. The initial engagement was defined as the proportion of individuals who completed the assessment of readiness and priority for lifestyle change during the first email. Ongoing engagement was defined as completion of the assessment of readiness and priority for lifestyle the second time at 6 weeks following initial engagement. Participants completed the second assessment of readiness and priority for lifestyle change within 8 weeks from receiving the re-assessment email. Characteristics of participants were extracted from self-reported data in the Heart&Stroke Risk Assessment, which is an online self-assessment questionnaire. Characteristics included age, gender, education level, ethnicity, employment, medical condition(s), and body mass index (calculated from height and weight). Modifiable CVD risk factors were defined as the following: physical activity level (whether participants achieved 30-60 minutes of moderate exercise, 4 times per week), smoking status (Yes or No), excess alcohol consumption (Male: consumed >2 drinks a day or >14 drinks a week; Female: consumed >2 drinks a day, >9 drinks a week), and whether participants consumed high fat foods (3 or more times per week), fruits and vegetable (5 or more servings per day), and food with high salt content (3 or more times per week).

### Data Analysis

Chi-square and independent *t* tests were used to compare characteristic differences between the Conditioned Reward and Control groups. Statistical significance can be influenced by a large sample size [21]; therefore, we calculated effect size using Cramer’s *V* (post chi-square statistic) and Cohen’s *d* (post independent *t* test) to determine the strength of these statistically significant results. A weak, moderate, and strong association (effect size) for Cramer’s *V* was defined as <0.2, 0.2-0.6, and >0.6, respectively [22]. Meanwhile, a small, moderate, and large effect size for Cohen’s *d* was defined as ≤0.2, 0.5, and ≥0.8, respectively [23]. Binary logistic regressions were conducted to estimate the association between loyalty rewards and enrollment (model 1), initial engagement (model 2), and ongoing engagement (model 3) of the Internet-based health program. Odds ratios were calculated to identify the effects of loyalty rewards on the likelihood of enrolling and engaging with the My Health eSupport program. Based on previous research [11,18], all models were adjusted for age, ethnicity, education, gender, employment status, and the number of modifiable risk factors. Data were analyzed using SPSS version 19 and significance was accepted at an alpha level of .01.

## Results

### Participants

A total of 73,327 individuals in the Conditioned Reward group and 69,399 individuals in the Control group completed the Heart&Stroke Risk Assessment. Baseline characteristics of the participants are presented in Table 1. Overall, both groups attracted a larger proportion of females than males (68.48%, 97,732/142,726 vs. 31.52%, 44,994/142,726). Most of the participants were Caucasian (83.54%, 119,239/142,726). The most common illnesses in both groups were hypertension (22.43%, 32,014/142,726) and dyslipidemia (17.86%, 25,487/142,726). More than 50% of individuals reported having a BMI higher than 25. Two of the most common lifestyle factors associated with CVD risk for both groups were physical inactivity (44.10%, 62,942/142,726) and inadequate fruit and vegetable consumption (43.32%, 61,828/142,726) (Table 2).

**Table 1:** Baseline participant characteristics.

Characteristics		Conditioned Rewardn=73,327 n (%)	Controln=69,399 n (%)	*P* value	Effect size
Age (years), mean (SD)		50.4 (14.2)	50.5 (14.4)	.54	0.003
Female		49,778 (67.88%)	47,954 (69.11%)	<.001	0.01
Completed university education		43,783 (60.74%)	41,208 (60.53%)	.40	0.002
**Ethnicity**				<.001	0.13
	Caucasian	62,658 (85.48%)	56,581 (81.62%)		
	South Asian	1688 (2.30%)	1911 (2.76%)		
	Chinese	3040 (4.15%)	1236 (1.78%)		
	Aboriginal	1038 (1.42%)	1253 (1.81%)		
	African/black	683 (0.93%)	1160 (1.67%)		
**Marital status**				<.001	0.03
	Married	42,396 (57.85%)	40365 (58.18%)		
	Widowed, divorced	10,177 (13.89%)	8423 (12.14%)		
	Single	10,787 (14.72%)	10,397 (14.99%)		
**Employment**					
	Full or part-time	41,279 (56.33%)	40,345 (58.16%)	<.001	0.07
	Self-employed	6675 (9.11%)	6726 (9.70%)		
	Retired	14,644 (19.98%)	11,654 (16.80%)		
	Stay-at-home parent	3049 (4.16%)	2350 (3.39%)		
**Type of employment**				<.001	0.08
	Management/white collar	45,660 (63.00%)	43,364 (68.81%)		
	Sales or services	9491 (13.09%)	6757 (10.72%)		
	Trades	6408 (8.84%)	6082 (9.65%)		
**Body Mass Index**				<.001	0.13
	Underweight (<18.5)	1464 (2.00%)	1109 (1.60%)		
	Normal weight (18.5-24.9)	21,942 (29.93%)	19,384 (27.94%)		
	Overweight (25-29.9)	21,240 (28.97%)	22,356 (32.21%)		
	Obesity (≥30)	16,459 (22.45%)	20,039 (28.88%)		
**Mean number of cardiovascular co-morbidities (SD)**		0.54 (0.93)	0.51 (0.89)	<.001	0.03
	Diabetes	5298 (7.23%)	3072 (4.43%)	<.001	0.06
	Dyslipidemia	13,984 (19.07%)	11,503 (16.58%)	<.001	0.03
	Heart disease	2795 (3.81%)	2662 (3.84%)	.81	0.001
	Hypertension	15,520 (21.17%)	16,494 (23.77%)	<.001	0.11
	Stroke	1254 (1.71%)	1327 (1.91%)	.004	0.01
	Mood disorder	11,906 (16.24%)	8857 (12.76%)	<.001	0.05
	Sleep apnea	3988 (5.44%)	3065 (4.42%)	<.001	0.02

**Table 2:** Baseline modifiable risk factors.

Modifiable risk factors	Conditioned Reward n=73,327 n (%)	Control n=69,399 n (%)	*P* value	Effect size
Physical inactivity	30,972 (42.24%)	31,970 (46.07%)	<.001	0.04
Smoker	5704 (8.22%)	8270 (11.30%)	<.001	0.05
Excess alcohol	17,095 (23.31%)	16,615 (23.94%)	.005	0.01
Fatty foods	8537 (11.69%)	10,663 (15.42%)	<.001	0.06
Infrequent fruit and vegetable	30,601 (41.82%)	31,227 (45.05%)	<.001	0.03
Salt	19,738 (26.92%)	14,205 (20.47%)	<.001	0.08
Mean number of modifiable risk factors (SD)	2.51 (1.37)	2.58 (1.37)	<.001	0.05

### Enrollment Rates

Enrollment in the My Health eSupport program was higher in the Conditioned Reward group (52.96%, 38,835/73,327) than the Controls (4.07%, 2826/69,399). Loyalty rewards provision was the strongest predictor for enrollment in the My Health eSupport program (OR 27.9, 95% CI 26.4-29.4; *P*<.001). Factors influencing enrollment are presented in Table 3.

**Table 3:** Prediction of enrollment and engagement.

Factors		Enrollment			Initial engagement			Ongoing engagement		
		OR	(95% CI)	*P* value	OR	(95% CI)	*P* value	OR	(95% CI)	*P* value
Loyalty rewards (Conditioned Reward vs Control)		27.9	(26.4-29.4)	<.001	0.79	(0.66-0.92)	.004	0.93	(0.68-1.26)	.662
Gender (female vs male)		0.96	(0.93-0.99)	.007	1.26	(1.17-1.36)	<.001	1.27	(1.09-1.46)	.002
**Age**										
	≤29 years	1.98	(1.82-2.15)	<.001	1.37	(0.95-1.97)	.085	1.49	(0.62-3.55)	.371
	30-39 years	1.69	(1.56-1.82)	<.001	2.32	(1.64-3.27)	<.001	1.99	(0.86-4.57)	.104
	40-49 years	1.80	(1.66-1.94)	<.001	4.41	(3.14-6.17)	<.001	5.86	(2.63-13.0)	<.001
	50-59 years	1.80	(1.66-1.94)	<.001	6.81	(4.87-9.49)	<.001	9.45	(4.27-20.8)	<.001
	≥60 years	2.12	(1.96-2.30)	<.001	8.40	(6.00-11.7)	<.001	12.56	(5.66-27.8)	<.001
Ethnicity (Caucasian vs other)		1.20	(1.15-1.25)	<.001	1.13	(1.01-1.24)	.025	1.12	(0.89-1.38)	.318
Total number of modifiable risk factors		0.96	(0.95-0.97)	<.001	1.37	(1.33-1.40)	<.001	1.38	(1.31-1.45)	<.001
Employment (employed vs not employed)		1.02	(0.99-1.06)	.201	0.89	(0.82-0.95)	<.001	1.03	(0.88-1.20)	.676
University education (completed vs not completed)		1.05	(1.01-1.08)	.004	0.86	(0.79-0.91)	<.001	0.88	(0.76-1.01)	.068

### Engagement Level

After participants enrolled in the My Health eSupport program, only 12.43% (4829/38,835) of individuals in the Conditioned Reward group and 8.49% (240/2826) of the Controls assessed their readiness for change and selected a priority area for lifestyle change. Out of these participants, 20.98% (1013/4829) in the Conditioned Reward group and 24.17% (58/240) in the Control group completed the second assessment at week 6. In our regression analyses, loyalty rewards strategy was negatively associated with initial engagement (OR 0.79, 95% CI 0.66-0.92; *P*=.004) and it was not a significant predictor for ongoing engagement ( *P*=.66). Meanwhile, age, gender, and numbers of modifiable risk factors were significant predictors of ongoing engagement (Table 3).

## Discussion

### Principal Findings

The main finding of this study was that a single exposure of loyalty rewards significantly influenced enrollment for the My Health eSupport program. Individuals were 27.9 times more likely to enroll when presented with loyalty rewards. However, contrary to our hypothesis, ongoing engagement was not influenced by loyalty rewards. Individuals were more likely to engage with the program if they were greater than 60 years of age, female, or had one or more modifiable risk factors. These findings suggest that a single exposure of loyalty rewards may be used to encourage individuals to sign up for an Internet-based preventative health initiative, but supplemental strategies are required to maintain engagement.

Our findings on the effects of loyalty rewards with online enrollment were consistent with other study findings using financial rewards [24]. Alexander et al [18] reported that financial incentives of $2.00, $5.00, and $10.00 offered at the point of enrollment were able to enroll 3.5%, 7.5%, and 2.7% of the participants, respectively. It is encouraging to observe that, in this study, 20 Air Miles (equivalent to about $2.00 CAD) was sufficient to enroll approximately 50% of the participants. It is important to take into consideration that the participants in the Conditioned Reward group were from the Air Miles loyalty reward program. These individuals may have perceived the value of the loyalty reward as being greater than the direct monetary value of $2.00. As a result, our findings may be limited in the ability to generalize beyond Air Miles members.

Engagement level with Internet-based interventions has always been a challenge as high dropout and losses to follow-up are common. This phenomenon is described by Eysenbach as “the law of attrition” [25]. It is hypothesized that the attrition curve can be a valuable marker of the underlying causes for attrition. In our study, the attrition followed an L-shaped curve, which reflected an initial rapid decline of participants, with a remaining group of steady “hardcore” users who continuously used the system over the 6-week interval of this study. This type of attrition curve may indicate that the enrolled participants may not be the appropriate user group for the HSFC website [25] and/or that participants did not perceive the benefits of continued engagement with the Internet-based health intervention [26]. Since the My Health eSupport program was designed to reduce CVD risk through lifestyle changes, it may not be surprising that the minority of individuals who continued to engage with the eSupport program were those with higher CVD risk, such as older individuals or individuals with one or more modifiable CVD risk factors.

A single exposure of loyalty reward may have also contributed to the low level of engagement with the My Health eSupport program. Since rewards were presented at the point of enrollment, some individuals in the Conditioned Reward group may have enrolled for the sole purpose of obtaining this reward. This may explain the negative association found between loyalty rewards and initial engagement level. Charness et al [27] found that financial rewards may need to be presented on a number of occurrences (for at least 5 weeks) in order to help individuals pass the “threshold” level needed to sustain an activity on their own for up to 16 weeks. The long-term effectiveness (>6 months) of using financial incentives to maintain behavior change remains unclear, primarily due to the fact that the majority of studies are short term (<6 months) [17]. A recent meta-analysis reported several design features for financial rewards that may help increase adherence to exercise programs. These included (1) providing an assured financial incentive rather than lottery or chance-based reward, (2) offering larger incentives, and (3) incorporating incentives that are designed to be indexed (eg, $25/pound of weight loss), or escalating (eg, $10 for the first pound lost, $15 for second pound lost, etc) [17]. It is important to incorporate these design features into future studies aimed at behavior change using loyalty rewards.

Some researchers have cautioned against the use of any external incentives as they may have the undesired effects of inhibiting intrinsic motivation [27,28]. Future studies on Internet-based interventions should consider how indexed or escalated loyalty rewards could be used to sustain program engagement while maintaining or increasing intrinsic motivation associated with a health behavior change. Based on self-determination theory, intrinsic motivation is fulfilled by the psychological needs of competence (a sense of mastery), autonomy (ownership over behavior), and social relatedness (feel socially connected to others) [29]. Mitchell et al suggested that extrinsic rewards may be used to fulfill these psychological needs in order to avoid harming intrinsic motivation by (1) rewarding achievements of realistic self-regulatory goals (eg, the use of self-monitoring), (2) providing choice for the types of reward and the activities chosen, and (3) offering rewards that are related to social outcomes (eg, group contingencies or charitable donations) [17]. Additionally, it may be necessary to combine loyalty rewards with previously established key components of counselling in order to maintain levels of engagement in Internet-based interventions. These components include: goal setting and self-monitoring; the provision of feedback based on the patients’ activity; self-efficacy enhancement; and relapse prevention [30].

### Limitations and Strengths

Several limitations of the present study should be noted. The accuracy of self-reported data is open to challenge for validity. Self-report bias such as lifestyle behaviors of exercise, diet, and smoking could have influenced the accuracy of our results. Engagement with the system was defined when participants re-assessed their readiness for change and selected another priority area for lifestyle change. It is possible that individuals maintained engagement with the program but never completed the re-assessment, which may underestimate the levels of engagement with the My Health eSupport Program. There are other variables that may be associated with levels of enrollment and engagement which were not assessed, such as income, anxiety, and depression. Finally, there may be selection bias in our study as the Conditioned Reward group consisted of participants from the Air Miles loyalty reward program. This may limit our ability to generalize our findings beyond Air Miles members. A strength of this study includes the large sample size. This is one of the first “real-world” (eg, population-based) studies that has been conducted to examine the effects of loyalty rewards on enrollment and engagement with an Internet-based intervention.

### Conclusions

Internet-based interventions hold great potential for delivering preventive health initiatives at a population level. A single exposure of loyalty rewards can increase enrollment but additional strategies are required to maintain engagement level. This study has significant design implications for incorporating loyalty rewards as an effective enrollment strategy for future Internet-based interventions. More research is needed that explores the long-term effects of using loyalty rewards to reinforce engagement level and the associated efficacy of Internet-based health programs.

## Abbreviations

CVD: cardiovascular disease
HSFC: Heart and Stroke Foundation of Canada

## References

[1] Mathers CD, Loncar D (2006). Projections of global mortality and burden of disease from 2002 to 2030. PLoS Med.

[2] Buttar HS, Li T, Ravi N (2005). Prevention of cardiovascular diseases: Role of exercise, dietary interventions, obesity and smoking cessation. Exp Clin Cardiol.

[3] (2013). Statistics Canada.

[4] Vandelanotte C, Spathonis KM, Eakin EG, Owen N (2007). Website-delivered physical activity interventions a review of the literature. Am J Prev Med.

[5] Yoo HJ, Park MS, Kim TN, Yang SJ, Cho GJ, Hwang TG, Baik SH, Choi DS, Park GH, Choi KM (2009). A ubiquitous chronic disease care system using cellular phones and the internet. Diabet Med.

[6] Kuhl EA, Sears SF, Conti JB (2006). Internet-based behavioral change and psychosocial care for patients with cardiovascular disease: a review of cardiac disease-specific applications. Heart Lung.

[7] Liu S, Dunford SD, Leung YW, Brooks D, Thomas SG, Eysenbach G, Nolan RP (2013). Reducing blood pressure with Internet-based interventions: a meta-analysis. Can J Cardiol.

[8] Nolan RP, Liu S, Shoemaker JK, Hachinski V, Lynn H, Mikulis DJ, Wennberg RA, Moy Lum-Kwong M, Zbib A (2012). Therapeutic benefit of internet-based lifestyle counselling for hypertension. Can J Cardiol.

[9] Bennett GG, Herring SJ, Puleo E, Stein EK, Emmons KM, Gillman MW (2010). Web-based weight loss in primary care: a randomized controlled trial. Obesity (Silver Spring).

[10] Bennett GG, Glasgow RE (2009). The delivery of public health interventions via the Internet: actualizing their potential. Annu Rev Public Health.

[11] Stopponi MA, Alexander GL, McClure JB, Carroll NM, Divine GW, Calvi JH, Rolnick SJ, Strecher VJ, Johnson CC, Ritzwoller DP (2009). Recruitment to a randomized web-based nutritional intervention trial: characteristics of participants compared to non-participants. J Med Internet Res.

[12] Glasgow RE, Nelson CC, Kearney KA, Reid R, Ritzwoller DP, Strecher VJ, Couper MP, Green B, Wildenhaus K (2007). Reach, engagement, and retention in an Internet-based weight loss program in a multi-site randomized controlled trial. J Med Internet Res.

[13] Jeffery RW (2012). Financial incentives and weight control. Prev Med.

[14] Hunter RF, Tully MA, Davis M, Stevenson M, Kee F (2013). Physical activity loyalty cards for behavior change: a quasi-experimental study. Am J Prev Med.

[15] Rice T (2013). The behavioral economics of health and health care. Annu Rev Public Health.

[16] Teixeira PJ, Palmeira AL, Vansteenkiste M (2012). The role of self-determination theory and motivational interviewing in behavioral nutrition, physical activity, and health: an introduction to the IJBNPA special series. Int J Behav Nutr Phys Act.

[17] Mitchell MS, Goodman JM, Alter DA, John LK, Oh PI, Pakosh MT, Faulkner GE (2013). Financial incentives for exercise adherence in adults: systematic review and meta-analysis. Am J Prev Med.

[18] Alexander GL, Divine GW, Couper MP, McClure JB, Stopponi MA, Fortman KK, Tolsma DD, Strecher VJ, Johnson CC (2008). Effect of incentives and mailing features on online health program enrollment. Am J Prev Med.

[19] Prochaska JO, Velicer WF, Rossi JS, Goldstein MG, Marcus BH, Rakowski W, Fiore C, Harlow LL, Redding CA, Rosenbloom D (1994). Stages of change and decisional balance for 12 problem behaviors. Health Psychol.

[20] Miller WR, Rollnick S (2002). Motivational Interviewing: preparing people for change.

[21] Thomas JR, Nelson J, Silverman S (2010). Research Methods in Physical Activity - 6th Edition.

[22] Rea LM, Parker RA (1992). Designing and conducting survey research: a comprehensive guide.

[23] Cohen J (1994). The Earth is round (p < .05). American Psychologist.

[24] Taitel MS, Haufle V, Heck D, Loeppke R, Fetterolf D (2008). Incentives and other factors associated with employee participation in health risk assessments. J Occup Environ Med.

[25] Eysenbach G (2005). The law of attrition. J Med Internet Res.

[26] Rogers MA (2003). Diffusion of Innovations. 5 ed.

[27] Charness G, Gneezy U (2009). Incentives to exercise. Econometrica.

[28] Daley DJ, Duda JL (2006). Self-determination, stage of readiness to change for exercise, and frequency of physical activity in young people. Eur. J. Sport Sc.

[29] Mitchell M, Faulkner G (2012). A "nudge" at all? The jury is still out on financial health incentives. Healthc Pap.

[30] Artinian NT, Fletcher GF, Mozaffarian D, Kris-Etherton P, Van Horn L, Lichtenstein AH, Kumanyika S, Kraus WE, Fleg JL, Redeker NS, Meininger JC, Banks J, Stuart-Shor EM, Fletcher BJ, Miller TD, Hughes S, Braun LT, Kopin LA, Berra K, Hayman LL, Ewing LJ, Ades PA, Durstine JL, Houston-Miller N, Burke LE, American Heart Association Prevention Committee of the Council on Cardiovascular Nursing (2010). Interventions to promote physical activity and dietary lifestyle changes for cardiovascular risk factor reduction in adults: a scientific statement from the American Heart Association. Circulation.

